# Kleptoplast photosynthesis is nutritionally relevant in the sea slug *Elysia viridis*

**DOI:** 10.1038/s41598-017-08002-0

**Published:** 2017-08-10

**Authors:** Paulo Cartaxana, Erik Trampe, Michael Kühl, Sónia Cruz

**Affiliations:** 1 Departamento de Biologia & Centro de Estudos do Ambiente e do Mar (CESAM), Universidade de Aveiro, Aveiro, Portugal; 2 Marine Biological Section, Department of Biology, University of Copenhagen, Helsingør, Denmark; 3 Climate Change Cluster, University of Technology Sydney, Sydney, NSW Australia

## Abstract

Several sacoglossan sea slug species feed on macroalgae and incorporate chloroplasts into tubular cells of their digestive diverticula. We investigated the role of the “stolen” chloroplasts (kleptoplasts) in the nutrition of the sea slug *Elysia viridis* and assessed how their abundance, distribution and photosynthetic activity were affected by light and starvation. *Elysia viridis* individuals feeding on the macroalga *Codium tomentosum* were compared with starved specimens kept in dark and low light conditions. A combination of variable Chl *a* fluorescence and hyperspectral imaging, and HPLC pigment analysis was used to evaluate the spatial and temporal variability of photopigments and of the photosynthetic capacity of kleptoplasts. We show increased loss of weight and body length in dark-starved *E*. *viridis* as compared to low light-starved sea slugs. A more pronounced decrease in kleptoplast abundance and lower photosynthetic electron transport rates were observed in dark-starved sea slugs than in low light-starved animals. This study presents strong evidence of the importance of kleptoplast photosynthesis for the nutrition of *E*. *viridis* in periods of food scarcity. Deprived of photosynthates, *E*. *viridis* could accelerate the breakdown of kleptoplasts in the dark to satisfy its’ energy requirements.

## Introduction

Mixotrophic organisms are able to obtain organic carbon via both heterotrophic and phototrophic metabolisms^1^, and include several sacoglossan sea slug species that use their radular teeth to penetrate the cell wall of algal filaments, suck and digest the cellular content, while incorporating functional algal chloroplasts into tubular cells of their digestive diverticula via a process known as kleptoplasty^2, 3^. These acquired chloroplasts – commonly termed kleptoplasts – do not divide^4^, but may remain functional for periods ranging from a few days to several months^5, 6^. The incorporation and maintenance of functional chloroplasts into animal tissues constitutes one of the most puzzling natural photosynthetic systems due to the absence of the original nucleus and the fact that the chloroplast genome encodes only a small fraction of the proteins considered necessary for photosynthesis^7^.

Early studies using the sea slug *Elysia viridis* demonstrated the incorporation of photosynthetically fixed ^14^C in low-molecular-weight metabolites such as glucose, galactose, or amino acids^8^. The sea slug *E*. *chlorotica* maintains photosynthetic O_2_ evolution for several months in the absence of any external algal food supply^9^. Photosynthesis studies using light-driven ^14^CO_2_ incorporation or O_2_ evolution in sacoglossan sea slugs have been replaced to a large extent by variable chlorophyll (Chl) *a* fluorescence measurements using Pulse Amplitude Modulated (PAM) fluorimetry^5, 6, 10, 11^. Although capable of real-time, rapid and non-intrusive measurements following the same specimens over time, variable Chl *a* fluorescence measurements have limitations that may lead to erroneous interpretations^12^. The above mentioned studies used single-point measurements of variable Chl *a* fluorescence and did not consider spatial heterogeneities. In recent years, new imaging techniques such as Chl *a* fluorescence and hyperspectral imaging have been used to map pigment content and photosynthetic activity in microbial phototrophs and symbioses^13–15^ as well as kleptoplastidic dinoflagellates^16^. Such techniques now allow the assessment of spatial differences on scales ranging from the single-cell to areas of several square centimeters, improving our capacity to understand spatial and functional relationships.

Although acquired photosynthesis from kleptoplasts alone cannot support growth in sacoglossan sea slugs under laboratory conditions, most studies have identified nutritional benefits through periods of food scarcity^17–19^. The importance of photosynthesis for the nutrition of kleptoplastidic sea slugs is typically addressed by comparing growth parameters and survival rates of starved specimens (deprived of exogenous food sources, and therefore unable to feed heterotrophically) kept in the laboratory under dark (thus unable to acquire photosynthates from their kleptoplasts) and light conditions. A more pronounced decrease in weight/size and/or lower survival rates of specimens kept in the dark have been reported for *E*. *viridis*, *E*. *timida*, *E*. *chlorotica* and *Plakobranchus ocellatus* ^17–20^. Recently, Christa *et al*.^21^ found comparable weight loss in the sea slug *P*. *ocellatus* kept under dark and light conditions. Although the authors found incorporation of ^14^C to be severely reduced in the dark or in the presence of the photosynthesis inhibitor monolinuron, they advocate that kleptoplasts are retained exclusively as a source of stored food reserves. Hence, the effective contribution of acquired photosynthesis to the nutrition of sacoglossan sea slugs is still questioned in recent literature.

In this study, we investigated the role of kleptoplasts in the nutrition of the sacoglossan sea slug *E*. *viridis* and assessed how their abundance, distribution and photosynthetic activity were affected by light and starvation. *E*. *viridis* individuals feeding on the macroalga *Codium tomentosum* were compared with starved specimens kept in dark and low light conditions. A combination of hyperspectral imaging, variable Chl *a* fluorescence imaging, and pigment analysis with High Performance Liquid Chromatography (HPLC) was used to evaluate the spatial and temporal variability of photopigments and photosynthetic capacity of kleptoplasts. We addressed the following questions: i) Does blocking of photosynthesis through light deprivation in the absence of their algal food source increase the loss of sea slug body weight and length? ii) Are kleptoplasts of *E*. *viridis* kept in the dark as photosynthetically competent as kleptoplasts maintained under light/dark cycles? iii) How is kleptoplast abundance and distribution in *E*. *viridis* affected by starvation and light/dark conditions?

## Results

**Figure 1:**
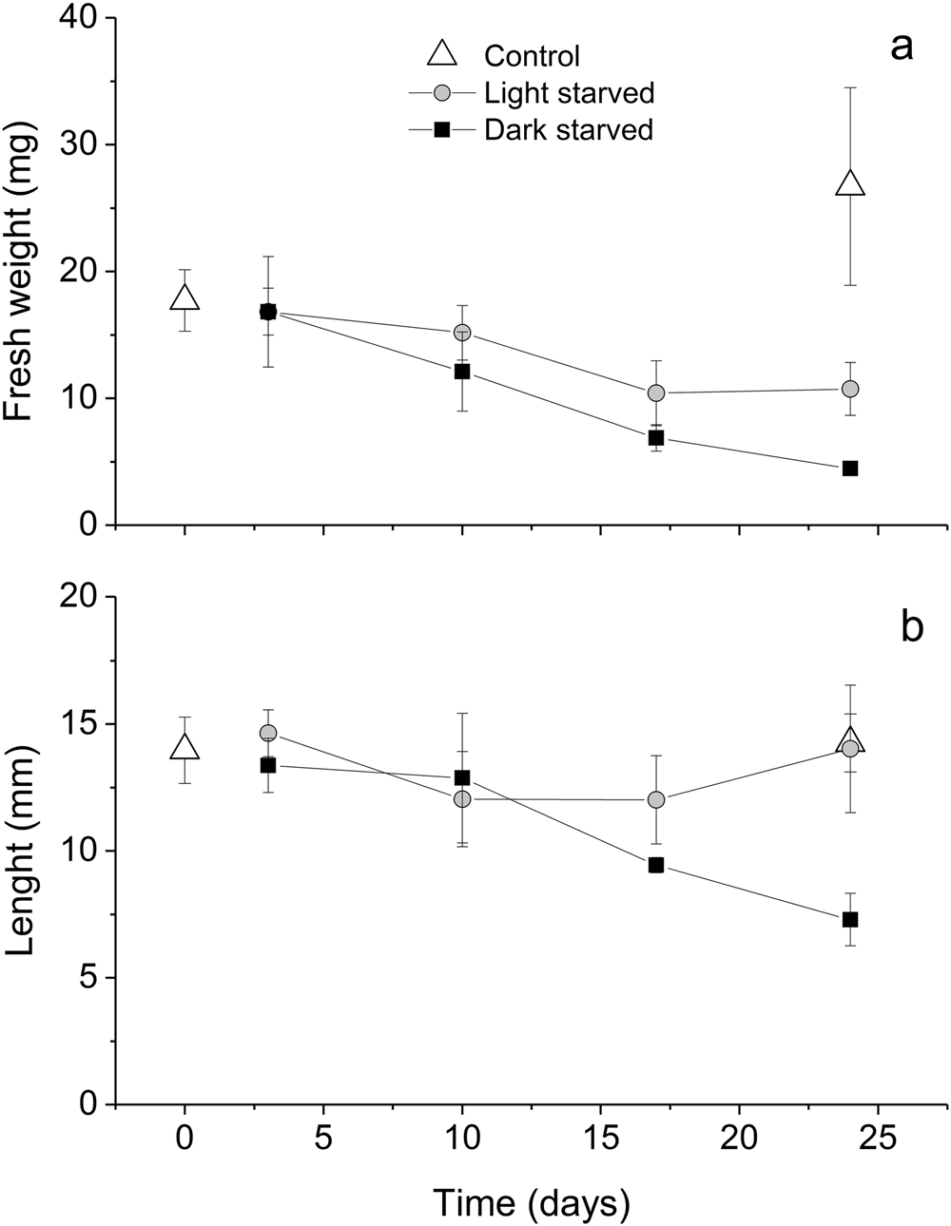
Changes in growth parameters of *Elysia viridis* with light and starvation. Fresh weight (**a**) and length (**b**) (mean ± standard deviation, n = 3) of animals feeding on *Codium tomentosum* (control) and starved under 14:10 h light:dark cycle (light-starved) and continuous darkness (dark-starved).

There was a significant effect of light treatment during starvation on both fresh weight and sea slug length (F_1,16_ = 10.154, p = 0.006; F_1,16_ = 12.937, p = 0.002, respectively; Fig. 1). Days of starvation also had a significant effect on both analyzed growth parameters (F_3,16_ = 18.418, p < 0.001 and F_3,16_ = 5.950, p = 0.006 for fresh weight and animal length, respectively). No statistically significant differences were observed in the length of light-starved individuals over time (F_3,8_ = 1.680, p = 0.248). After 24 days of starvation, fresh weight and animal length were significantly higher in light-starved than in dark-starved specimens (p = 0.015 and p = 0.008, respectively; Fig. 1). Fresh weight was significantly higher in control than in 24-days light-starved specimens (p = 0.009; Fig. 1a), but no significant differences were found for animal length (p = 0.999; Fig. 1b).

**Figure 2:**
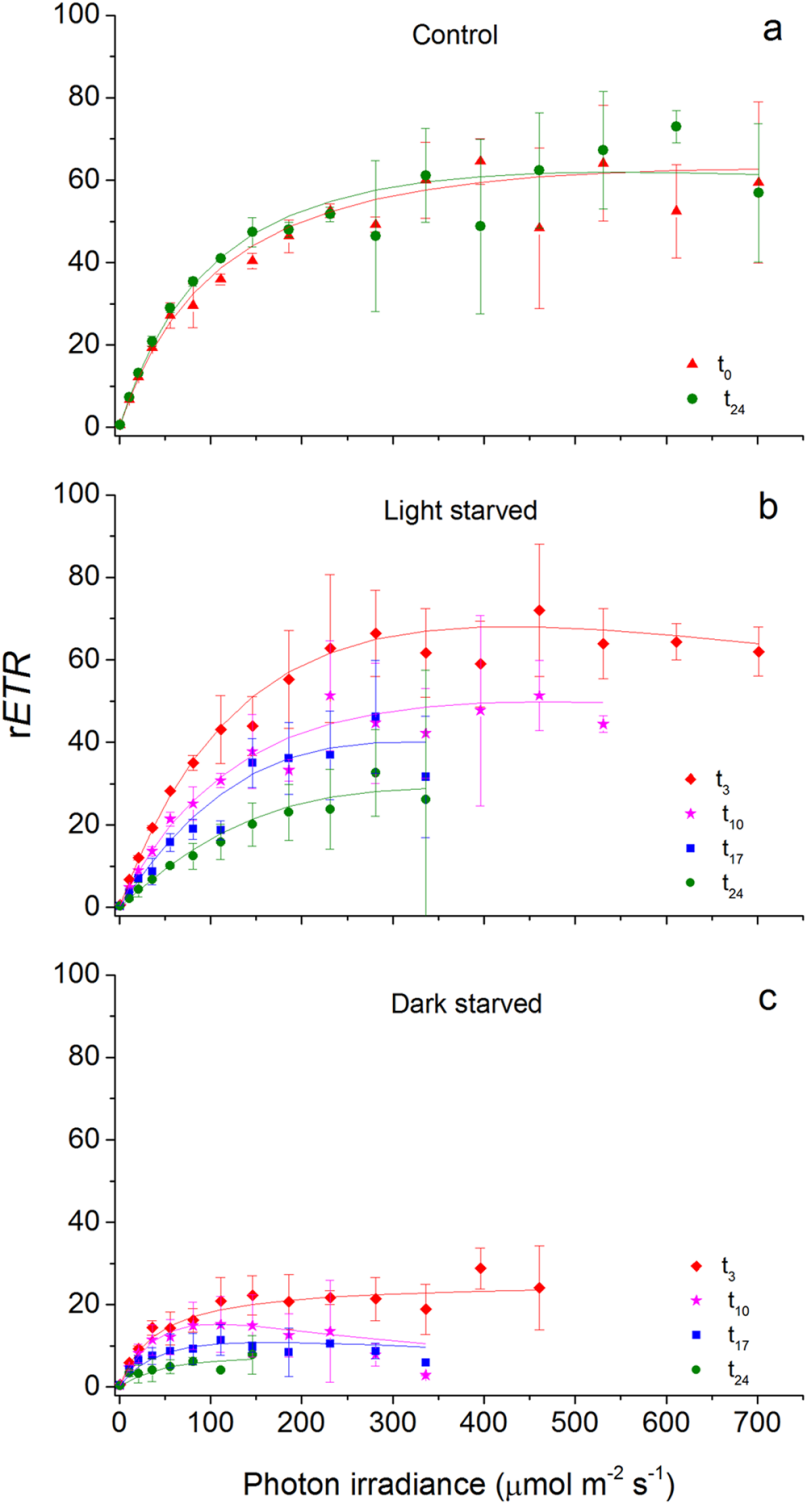
Rapid light-response curves of relative electron transport rate (r*ETR*) vs. photon irradiance in *Elysia viridis*. r*ETR* (mean ± standard deviation, n = 3) of animals feeding on *Codium tomentosum* (control, **a**), starved under a 14:10 h light:dark cycle (light-starved, **b**), and continuous darkness (dark-starved, **c**). The number of treatment days is represented as t_days_. Lines represent curve fits according to the model of Eilers and Peeters (1988).

**Figure 3:**
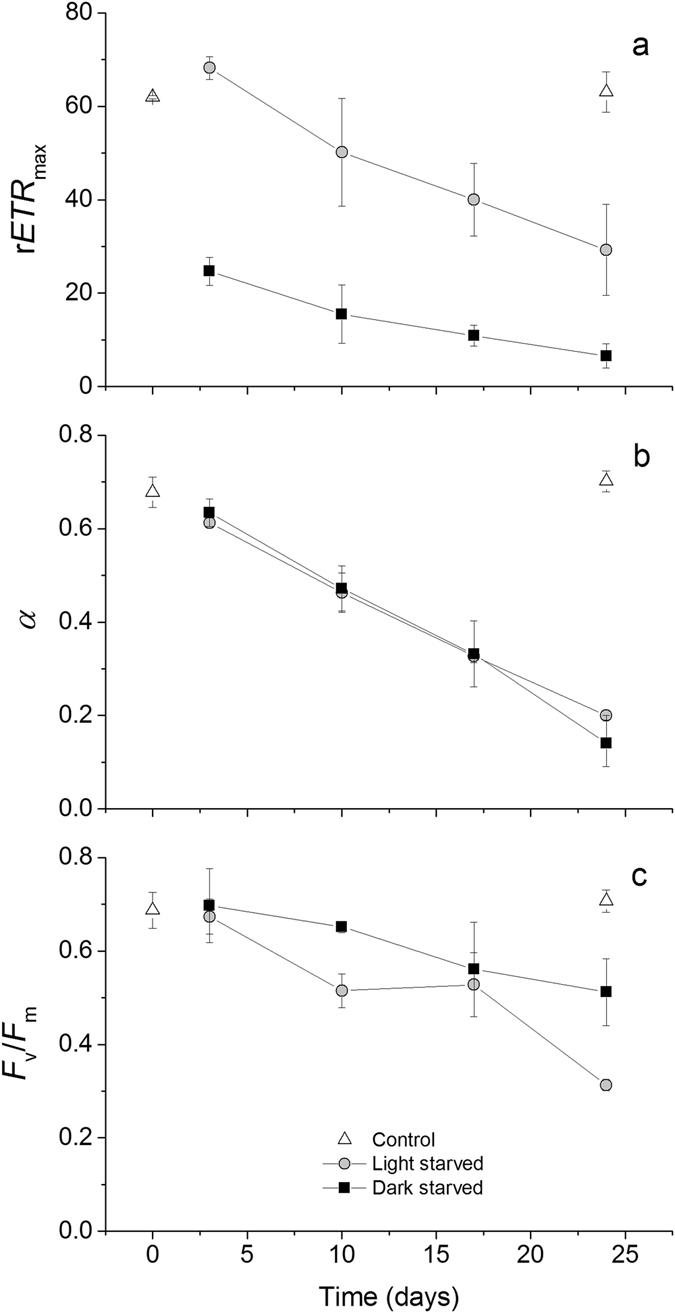
Changes in photosynthetic parameters of *Elysia viridis* with light and starvation. r*ETR*_max_ (relative maximum electron transport rate, **a**), *α* (light utilization coefficient, **b**), and *F*_v_/*F*_m_ (maximum quantum efficiency of PSII, **c**) (mean ± standard deviation, n = 3) of animals feeding on *Codium tomentosum* (control), starved under a 14:10 h light:dark cycle (light-starved) and continuous darkness (dark-starved).

Rapid light curve (RLC) measurements of the relative photosynthetic electron transport rate (r*ETR*) versus incident photon irradiance (*E*) on control (fed), light-, and dark-starved *E*. *viridis* are shown in Fig. 2. No differences were found in control individuals feeding on *C*. *tomentosum* (Fig. 2a). Days of starvation caused a clear decrease in r*ETR* measured in animals maintained under both light and dark conditions (Fig. 2b and c). However, r*ETR* values were comparatively higher in light-starved than in dark-starved *E*. *viridis*. Accordingly, there were significant effects of both independent variables (light treatment and days of starvation; F_1,16_ = 142.678, p < 0.001 and F_3,16_ = 19.535, p < 0.001, respectively) on the photosynthetic capacity at saturating irradiances (r*ETR*_max_; Fig. 3a). After 24 days, r*ETR*_max_ was significantly higher in control, intermediate in light-starved, and lower in dark-starved *E*. *viridis* (in all cases p ≤ 0.01; Fig. 3a).

There was a significant effect of days of starvation (F_3,16_ = 141.198, p < 0.001) but no significant effect of light treatment during starvation (F_1,16_ = 0.125, p = 0.727) on *α*, the RLC parameter related to the photosynthetic efficiency at limiting irradiances (Fig. 3b). After 24 days, *α* was significantly higher in control than in light-, and dark-starved *E*. *viridis* (in both cases p < 0.001). No significant difference in the light utilization coefficient was found between 24-days light- and dark-starved animals (p = 0.128; Fig. 3b). There were significant effects of both light treatment and days of starvation (F_1,16_ = 17.044, p = 0.001; F_3,16_ = 21.716, p < 0.001, respectively) on *F*_v_/*F*_m_, the maximum efficiency of PSII (Fig. 3c). After 24 days, *F*_v_/*F*_m_ was significantly higher in control, intermediate in dark-starved, and lower in light-starved *E*. *viridis* (in all cases p ≤ 0.004; Fig. 3c).

**Figure 4:**
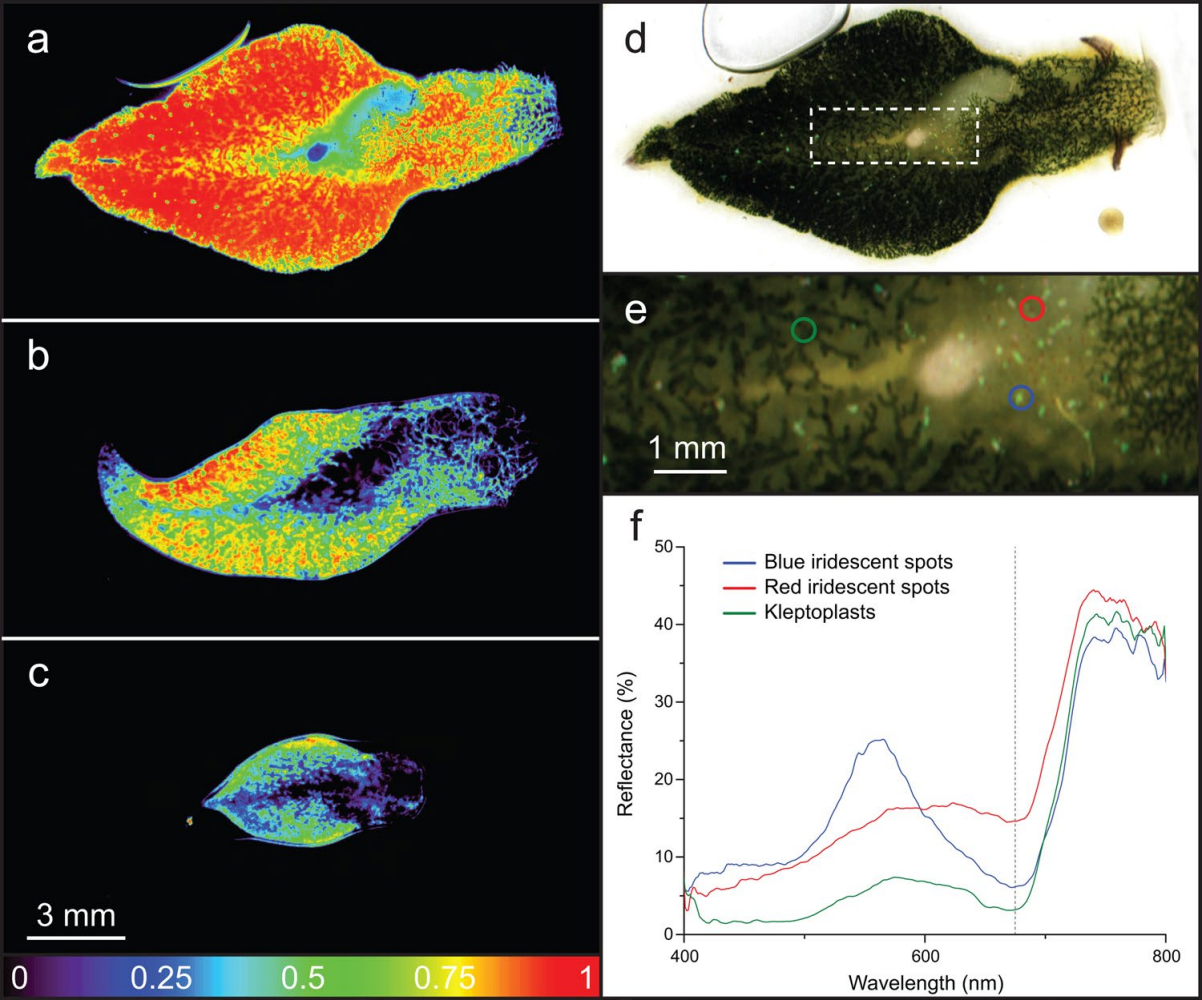
Hyperspectral imaging of *Elysia viridis*. Normalized difference vegetation index (NDVI) images of animals feeding on *Codium tomentosum* (**a**), and starved for 24 days under 14:10 h light:dark cycles (**b**) and in continuous darkness (**c**). RGB image representing “true” colors of the control after 24 days (**d**). Close-up of area represented in the white box of panel (d), with color-coded AOI’s for kleptoplasts, and red and blue iridescent spots (**e**), and extracted spectral characteristics seen in (**f**). Scale bar in panel (c) applies to the images in panels (a–d).

Hyperspectral imaging of *E*. *viridis* showed a heterogeneous distribution of kleptoplasts with higher density in the sea slugs’ lateral wing-like extensions or parapodia (Fig. 4). A lower kleptoplast density was found in the head and in the pericardium area. Kleptoplast abundance was affected by light treatment and days of starvation (Fig. 4a–c, Supplementary Fig. S1). After 24 days of treatment, NDVI in the control was notably higher (Fig. 4a), intermediate for light-starved sea slugs (Fig. 4b), and lower for dark-starved animals (Fig. 4c). The progressive decrease in kleptoplast density with days of treatment in light and dark-starved animals is depicted in Supplementary Fig. S1. The hyperspectral image stack of the control after 24 days is represented as an RGB composite image (Fig. 4d), and spectral signatures indicating the presence of Chl *a* with absorbance at 675 nm, and signatures of blue and red iridescent spots found in *E*. *viridis* are shown in Fig. 4e,f.

**Figure 5:**
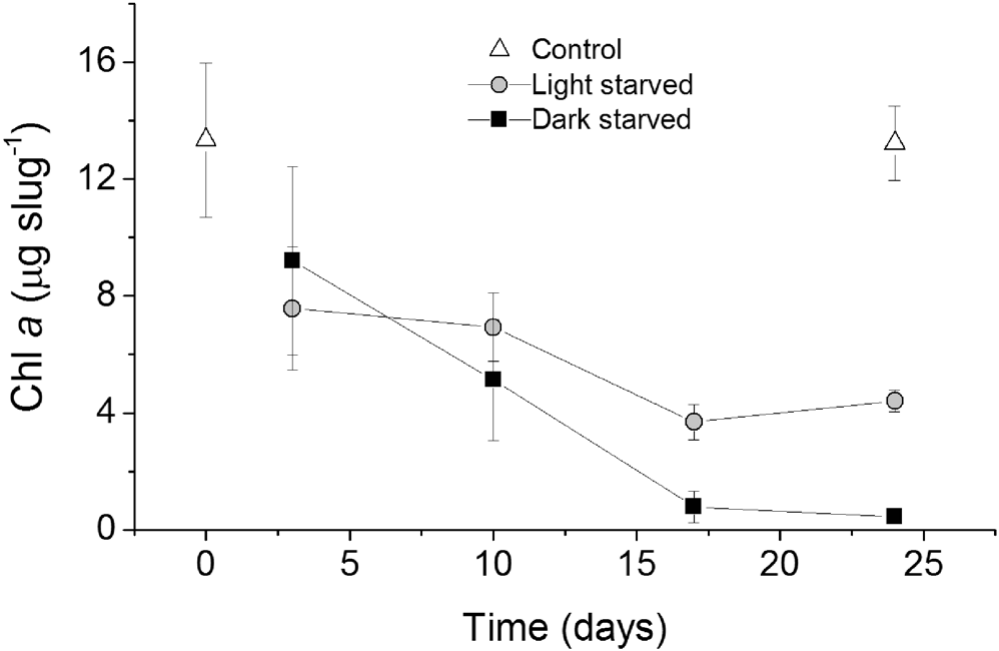
Changes in chlorophyll (Chl) *a* content of *Elysia viridis* with light and starvation. Chl *a* (mean ± standard deviation, n = 3) of animals feeding on *Codium tomentosum* (control), and starved under a 14:10 h light:dark cycle (light-starved) and in continuous darkness (dark-starved).

Pigments identified by HPLC in *E*. *viridis* included the carotenoids siphonaxanthin, *trans-* and *cis*-neoxanthin, violaxanthin, siphonaxanthin dodecenoate and β,ε-carotene, and the chlorophylls *a* and *b*. Concentrations of Chl *a* degradation products were residual representing in all cases <1% of Chl *a*. There were significant effects of both light treatment and days of starvation (F_1,16_ = 6.998, p = 0.018; F_3,16_ = 20.004, p < 0.001, respectively) on Chl *a* concentration per slug (Fig. 5). Although Chl *a* concentrations decreased markedly with starvation time, the decline was more pronounced for dark-starved animals. After 24 days, Chl *a* concentrations were significantly higher in control, intermediate in light-starved, and lower in dark-starved *E*. *viridis* (in all cases p ≤ 0.002; Fig. 5). Chl *a* concentrations expressed on a per weight basis confirmed the more pronounced decrease in kleptoplast abundance in dark-starved animals (Supplementary Table S1). Differences between control and 24-days light-starved animals (0.56 ± 0.17 and 0.42 ± 0.09 mg g^−1^ fw, respectively) were no longer significant (p = 0.356), whereas the 24-days dark-starved specimens had significant lower Chl *a* concentrations (0.10 ± 0.02 mg g^−1^ fw) than both the control and the 24-days light-starved animals (in both cases p < 0.05). Chl *a*/Chl *b* ratios were extremely stable during both light and dark starvation, while total carotenoids to Chls (Chl *a* + Chl *b*) ratios increased, particularly in dark-starved animals (Supplementary Table S1).

## Discussion

In this study, a more pronounced decrease in weight of starved *E*. *viridis* was observed when specimens were kept in the dark, unable to photosynthesize. Furthermore, a marked reduction in animal length was observed under starvation in the dark, whereas 24-days light-starved sea slugs showed comparable body length to individuals feeding on *C*. *tomentosum*. These results provide strong evidence for the importance of kleptoplast photosynthesis to the nutrition of *E*. *viridis* in periods of food scarcity. Similarly, Hinde & Smith^17^ found a more pronounced decrease in weight of *E*. *viridis* kept in the dark and argued that observed rates of photosynthesis were sufficient to account for the observed weight differences. Baumgartner *et al*.^22^ observed increased growth efficiency when *E*. *viridis* fed on *C*. *fragile*, due to photosynthesis by functional kleptoplasts, whereas feeding on *Cladophora rupestis* provided chloroplasts with highly limited functionality.

Acquired photosynthesis has also been found important for the nutrition of other sacoglossan sea slugs. Greater size loss and lower survival rates were reported for *E*. *timida*, *E*. *chlorotica* and *P*. *ocellatus* starved in the dark^18–20^. Using nitrogen isotope analysis of amino acids, Maeda *et al*.^23^ argued that although the nutritional role of photosynthesis in wild *P*. *ocellatus* feeding on multiple macroalgae species was negligible, kleptoplast photosynthates formed a significant source of nutrition for starved animals. Based on stable carbon isotope ratio data, Raven *et al*.^24^ estimated total carbon derived from kleptoplastic photosynthesis to range between 16 and 60% in several Australian sacoglossan species. Middlebrooks *et al*.^25^ observed an increase of foraging effort in 8- and 12-week-starved *E*. *clarki*, whereas this behavior was not recorded in 4-week-starved or satiated animals, in which the photosynthetic activity of kleptoplasts remained high. However, the nutritional benefits of acquired photosynthesis in periods of food scarcity are still questioned in sacoglossan sea slugs with functional kleptoplasts. Christa *et al*.^21^ found comparable weight loss in *P*. *ocellatus* starved under dark and light conditions and in the presence of monolinuron, an herbicide which inhibits photosynthesis. They also found that *F*_v_/*F*_m_ decreased similarly in both *E*. *timida* and *P*. *ocellatus* during starvation in the light or darkness and even in the presence of the photosynthesis inhibitor (*P*. *ocellatus* only). Therefore, Christa *et al*.^21^ concluded that “photosynthesis as a core carbon source cannot be essential for slug survival” and argued that kleptoplasts are slowly digestible food reserves. However, not only the sample size was particularly low for *P*. *ocellatus* measurements (2 individuals per treatment), but also the effects of the herbicide on the animals was not investigated. Furthermore, Christa *et al*.^21^ did not show a control treatment in the light while feeding for any of the two species. Without a light-feeding control it is not possible to access if the life-support conditions were adequate for animal maintenance (in the case of Christa *et al*.^21^, petri dishes). Inadequate life-support conditions can affect survival, weight and *F*_v_/*F*_m_ values. Although it is essential as a reassurance of animal well-being, a light-feeding control is often left out^5, 6, 18, 21^. In its absence, conclusions must be made cautiously since many other variables (e.g. water quality or temperature) could overcome specific treatment effects^12^.

In our study, kleptoplast photosynthetic activity as determined by variable chlorophyll fluorescence was maintained in *E*. *viridis* allowed to feed on *C*. *tomentosum*, while in starved animals photosynthesis declined with the duration of the starvation period for both light and dark conditions. When subjected to actinic light, kleptoplasts in dark-starved sea slugs showed significantly lower relative electron transport rates (r*ETR*) than in light-starved animals. Accordingly, photosynthetic capacity at saturating irradiances (r*ETR*_max_) was significantly higher in light-starved *E*. *viridis*. However, higher maximum quantum yield of PSII (*F*_v_/*F*_m_) was observed in dark-starved *E*. *viridis* than in light-starved specimens. Several factors could contribute to the maintenance of a higher *F*_v_/*F*_m_ while decreasing *ETR*. At wavelengths longer than 700 nm, PSI may contribute significantly to the variable chlorophyll fluorescence signal in leaves^26^. When exposed to different light conditions PSII to PSI ratios in the kleptoplasts could change, therefore contributing differently to the minimum fluorescence in the dark-acclimated state (*F*_o_) thus resulting in inaccurate information on PSII photosynthesis. However, we observed no changes in Chl *a*/Chl *b* ratios during starvation of *E*. *viridis*, suggesting no relative changes in the photosynthetic machinery components. Alternatively, in dark-starved specimens, *F*_v_/*F*_m_ values provide an estimation of PSII maximal quantum yield, while electron transport in the light depends also on the activity of all the other electron transporters and on availability of stromal electron acceptors^27^. These light reactions could have been affected by prolonged dark acclimation, leading to an over-reduction of the electron transport chain when dark-starved samples were exposed to light and therefore reducing *ETR*. Furthermore, recent works^11, 28^ showed that *C*. *tomentosum* chloroplasts do not activate the fast photoprotective response (qE component of non-photochemical quenching) so, although an acclimation period in dim light was applied, kleptoplasts in *E*. *viridis* could suffer from photoinhibition when exposed to high irradiance steps of light-response curves. This could explain why alpha was kept constant but r*ETR* decreased. Finally, *F*_v_/*F*_m_ values as high as 0.4 have also been observed in *C*. *tomentosum* previously frozen in liquid nitrogen and stored at −20 °C for 2 weeks (data not shown). When exposed to low actinic light, measurements of variable fluorescence in the frozen algae showed no r*ETR*. Hence, *F*_v_/*F*_m_ alone can be a poor indicator of photosynthetic competence of kleptoplasts. Our results stress the importance of measuring other variable chlorophyll fluorescence parameters apart from *F*_v_/*F*_m_ when investigating photosynthetic activity and kleptoplast longevity in sea slugs.

The robustness of the original algal chloroplasts and their capacity to deal with oxidative stress may be determinant for longer-term kleptoplast photosynthetic capacity in sacoglossan sea slugs^11, 29, 30^. Absorption of excessive light in photosynthetic organisms leads to increased production of reactive oxygen species (ROS) that can potentially cause photooxidative damage and inhibit photosynthesis, mainly through the inactivation of the PSII reaction center protein D1^31^. However, PSII repair mechanisms are necessary for phototrophs even under low light conditions, as significant degradation and re-synthesis of D1 protein under low irradiances have also been reported^32^. The incapacity of *E*. *viridis* to synthesize D1 or other key proteins involved in photosynthesis could explain the continuous loss of photosynthetic activity observed in our study under low light conditions, and also the premature loss of kleptoplasts exposed to high light^33, 34^. The capacity for *de novo* synthesis of D1 protein and other key proteins such as the large subunit of RuBisCO has been shown for *E*. *chlorotica* ^9, 35, 36^, the sea slug species displaying the longest retention of functional kletoplasty. ROS accumulation in the digestive tubules of starving *E*. *cornigera*, but not *E*. *timida*, was related to the shorter survival of the former species under starvation^30^. The presence of an active xanthophyll cycle capable of dissipating excessive light energy as heat has also been suggested to play a key role in kleptoplast retention in both *E*. *timida* and *E*. *chlorotica* ^11, 37^. However, this photoprotection mechanism is absent in *E*. *viridis* kleptoplasts, as well as in the original *C*. *tomentosum* chloroplasts^11, 28, 38^.

Kleptoplast distribution as revealed by hyperspectral imaging showed a higher density in the sea slug’s parapodia, where the digestive system is more ramified. Hyperspectral imaging and pigment analysis showed a more pronounced decrease in kleptoplast abundance in starved *E*. *viridis*, when specimens were kept in the dark. Hence, it is apparent that a mechanism of intracellular kleptoplast breakdown in *E*. *viridis* is more active under dark than light conditions. It is possible that the increased breakdown of kleptoplasts in dark-starved *E*. *viridis* is related to the absence of other sources of nutrition to satisfy the animal’s energy requirements. It was previously suggested by Christa *et al*.^21^ that kleptoplasts could constitute a source of stored food in *E*. *timida* and *P*. *ocellatus*. In a recent review, Pierce *et al*.^39^ criticize the hypothesis that kleptoplasts might serve as an emergency food supply in sacoglossan sea slugs and claimed that it is not supported by morphological studies on photodamaged, aged kleptoplasts of *E*. *chlorotica* that show an apoptotic-like process of the entire kleptoplast-containing cell^40^. However, Trench *et al*.^8^, studying kleptoplast ultrastructure in *E*. *viridis*, found many vacuoles in specimens of dark-starved animals that contained disorganized chloroplast membranes, suggesting that kleptoplasts were digested. Pelletreau *et al*.^19^, studying the establishment of permanent kleptoplasty in *E*. *cholorotica*, observed that photosynthates accumulated in the animal in the form of lipid droplets, presumably used as an energy source by the animal, leaving the kleptoplasts undisturbed in the animal cytosol. However, if lipid stores were depleted, as was observed in animals allowed to feed only for <5 days post-metamorphosis, kleptoplasts were targeted for breakdown and permanent kleptoplasty was not acquired. Laetz *et al*.^41^ showed an inverse relationship between functional chloroplasts and lysosome density in starved *E*. *viridis* and *E*. *timida*, indicating that functional digestion is indeed occurring and that lysosomes are involved in this breakdown. Following photosynthetic-derived starch accumulation and use in starved *E*. *timida*, Laetz *et al*.^42^ suggested that kleptoplasts function simultaneously as a nutritive producer and a storage device.

In conclusion, this study shows increased loss of weight and body length and decreased photosynthetic competence in dark-starved *E*. *viridis*. Furthermore, we observed decreased kleptoplast retention in starved *E*. *viridis* in the absence of light. Deprived of photosynthates, *E*. *viridis* could accelerate the breakdown of kleptoplasts in the dark to satisfy its’ energy requirements. This study thus presents evidence that photosynthesis is nutritionally relevant in the sacoglossan sea slug *E*. *viridis*. However, more research and new methodologies are essential to unequivocally establish the full role of acquired photosynthesis in sacoglossan sea slugs.

## Methods

### Sampling and Experimental Set-up

Specimens of the sea slug *Elysia viridis* Montagu, 1804 and of the macroalga *Codium tomentosum* Stackhouse, 1797 were collected in the intertidal rocky zone of Labruge Beach, Portugal (41°16′26.6″N, 8°43′47.5″W) in late September 2015. The sea slugs and macroalgae were maintained in filtered seawater with salinity of 35, temperature of 15 °C (according to temperature measured *in situ* during collection), and a 14 h light:10 h dark cycle at a photon irradiance (400–700 nm) of 30 µmol photons m^−2^ s^−1^. Animals were acclimated to these experimental conditions for 16 days, while feeding on the macroalgae. Thirty specimens of similar size were chosen for the experiment, three of which were randomly sampled to characterize the initial conditions (control t_0_). Another three animals were kept feeding during 24 days (control t_24_), while the remaining 24 specimens were divided between two starvation treatments (deprived from their food source, *C*. *tomentosum*): (i) light-starved (14 h light:10 h dark), and (ii) dark-starved (continuous dark). Previous observations showed that individuals fed in the dark were hardly ever found on the food source. It is likely the case that *E*. *viridis* in total darkness does not feed in the same way than in the presence of light. Therefore, a dark-fed treatment was not used. Animals used in the experiment were maintained in 200 mL independent beakers with daily changes of seawater. Three replicates were sampled from each starvation treatment after 3, 10, 17 and 24 days (t_3_, t_10_, t_17_ and t_24_), respectively.

### Variable Chlorophyll *a* Fluorescence

Variable Chl *a* fluorescence was measured using an imaging fluorometer (I-PAM, IMAG-MAX/L, Heinz Walz GmbH, Germany) employing a CCD camera with a 12.5 mm objective lens and a red high-power LED illumination unit that provided the measuring beam, the actinic light and the saturating light pulses. Measurements of variable Chl fluorescence were performed simultaneously on sampled *E*. *viridis* sea slugs. Each individual was placed in a separate well of a 2 mm-deep black well-plate, where each well was filled with seawater and covered with a glass slide. Using a mounting stand (IMAG-MAX/GS; Heinz Walz GmbH, Germany), the illumination unit and the CCD camera were coupled at a fixed working distance, resulting in homogenous illumination of the imaged area. Numerical values of variable chlorophyll fluorescence parameters were extracted from the digital images using the imaging system software (Imaging Win, Heinz Walz GmbH, Germany) by selecting areas of interest (AOI) corresponding to each slug for every saturating light pulse.

Maximum quantum yield of photosystem (PS) II (*F*_v_/*F*_m_) was determined by calculating (*F*_m_ − *F*_o_)/*F*_m_, where *F*_m_ and *F*_o_ are the maximum and the minimum fluorescence of dark-adapted samples, respectively^43^. Slugs exposed to light (control and light-starved) were dark-adapted for 15 min prior to *F*_v_/*F*_m_ measurements. A relative measure of photosynthetic activity was also assessed through rapid light curves (RLC)^44^ via measurements of the effective PSII quantum yield at increasing photon irradiance of actinic light: 11, 21, 36, 56, 81, 111, 146, 186, 231, 281, 336, 396, 461, 531, 611 and 701 µmol photons m^−2^ s^−1^. The duration of each irradiance step was 10 s. Slugs were exposed for 15 min to low light (11 µmol photons m^−2^ s^−1^) before the initiation of the RLC, which were constructed by calculating, for each level of actinic light, the relative electron transport rate (r*ETR*) at the delivered actinic photon irradiance (*E*), and the effective quantum yield of PSII (Δ*F*/*F*_m_′) as r*ETR* = *E* × (*F*_m_′ − *F)*/*F*_m_′, where *F*_m_′ and *F* are the maximum and the minimum fluorescence of light exposed samples, respectively. The light response was characterized by fitting the model of Eilers & Peeters^45^ to r*ETR* versus *E* curves and by estimating the initial slope of the light curve *α* (light utilization coefficient) and r*ETR*_max_ (maximum r*ETR*).

### Hyperspectral Imaging

Prior to hyperspectral imaging, sampled *E*. *viridis* individuals were anaesthetized with eugenol according to Cruz *et al*.^46^. Immobilized sea slugs were carefully positioned on a concave slide and the parapodia opened with the help of a spotter brush. The slug was then covered with a coverslip and imaged with a hyperspectral camera system (100T-VNIR, Themis Vision Systems, St. Louis, MO, USA)^14^ mounted on a dissection microscope via the C-mount (Olympus SZX16, Olympus Corporation, Tokyo, Japan). Illumination of the sample during image acquisition was provided by a fiber-optic halogen lamp with an annular ring-light (KL-2500, and Annular Ring-light, Schott AG, Mainz, Germany) fitted on the objective of the dissection microscope. For hyperspectral image analysis, a dark signal image stack with no light reaching the camera, a reference image stack with the hyperspectral system focused on an illuminated 20% reflectance standard (Spectralon SRM- 20, LabSphere Inc., North Sutton, New Hampshire, USA), and image stacks of the mounted sea slugs were recorded. The hyperspectral system imaging software (PhiLumnia Hyperspectral Imager V. 4.2, PhiLumina, LLC, Gulfport, MS, USA) was used to perform dark correction, and normalization of measured image stacks to the 20% reference image stack in order to obtain calibrated image stacks in % reflection. Normalized difference vegetation index (NDVI) was calculated pixel wise as $NDVI=\frac{{R}_{NIR}-\,{R}_{red}}{{R}_{NIR}+{R}_{red}}$ for each of the calibrated image stacks using the software package ENVI (Exelis Visual Information Solutions, Boulder, CO, USA), where ${R}_{{NIR}}$ and ${R}_{{red}}$ represent the reflectivity at band 258 (750 nm) and band 203 (675 nm), respectively. Animal length was determined from calibrated bright field images of each slug using the open source software ImageJ (version 1.49).

### Weight Measurements

Animal fresh weight was determined after Chl *a* fluorescence and hyperspectral imaging to reduce manipulation of *E*. *viridis* sea slugs before the measurements. The coverslips covering the animals were removed and excessive water eliminated with absorbing paper. Animals were then carefully removed from the concave slide using a spotter brush and placed in 1.5 mL plastic tubes for fresh weight measurements. Samples were immersed in liquid nitrogen and stored at −80 °C for subsequent pigment analyses.

### Pigment Analysis

Frozen *E*. *viridis* individuals were ground with a micro pestle and extracted in a mixture of acetone and methanol (7:2), where the samples were sonicated (S-4000, Branson Ultrasonic Corporation, USA) for 10 s to improve pigment extraction and centrifuged for 20 s at 13,400 rpm. Supernatants were filtered through 0.45 µm PTFE-membranes and immediately injected in a HPLC (1260 Infinity, Agilent Technologies, USA) equipped with a photodiode array detector. Prior to sample injection, 15 µL of 1 M ammonium acetate was added to each HPLC vial as a resolution-improving agent. The solvent gradient followed Frigaard *et al*.^47^ with a 69 min elution program, a flow rate of 1.0 mL min^−1^ and an injection volume of 100 μL. Chromatographic separation was carried out using a C18 Ascentis^®^ column for reverse phase chromatography (5 µm particle size; L × I.D.: 25 cm × 4.6 mm). Pigments were identified by comparison of retention times and absorbance spectra^48^. Concentrations of Chl *a* were calculated from the signals in the photodiode array detector calibrated using 4 dilutions of a commercially available pigment standard (Sigma-Aldrich, USA). Pigment ratios were calculated with the respective peak areas to evaluate relative changes on pigment composition during starvation.

### Statistical Analysis

Significance of differences in measured parameters was tested using two-way analysis of variance (ANOVA) for the effects of light treatments (14 h light:10 h dark, and continuous dark) and days of starvation (3, 10, 17 and 24 days). Significant differences between control (fed), 24-days light- and dark-starved animals were tested using one-way ANOVA. Multiple comparisons were performed using Tukey HSD. Statistical analyses were carried out using SPSS Statistics 22 (IBM, USA).

## Electronic supplementary material

Supplementary Information
