# Supplementary material for: Kleptoplast photosynthesis is nutritionally relevant in the sea slug Elysia viridis
Source: Sci Rep. 2017 Aug 10;7:7714. doi: 10.1038/s41598-017-08002-0 (PMC5552801; doi:10.1038/s41598-017-08002-0)
Supplement: Supplementary file 1 — Supplementary Information [file 41598_2017_8002_MOESM1_ESM.pdf]

Supplementary Information for:

Kleptoplast photosynthesis is nutritionally relevant in the sea slug *Elysia viridis*

Paulo Cartaxana[1], Erik Trampe[2], Michael Kühl[2,3], Sónia Cruz[1,*]

[1] Departamento de Biologia & Centro de Estudos do Ambiente e do Mar (CESAM), Universidade de Aveiro, Aveiro, Portugal

[2] Marine Biological Section, Department of Biology, University of Copenhagen, Helsingør, Denmark

[3] Climate Change Cluster, University of Technology Sydney, NSW, Australia

* Corresponding author: Tel: +351 234247092 ; Email: sonia.cruz@ua.pt

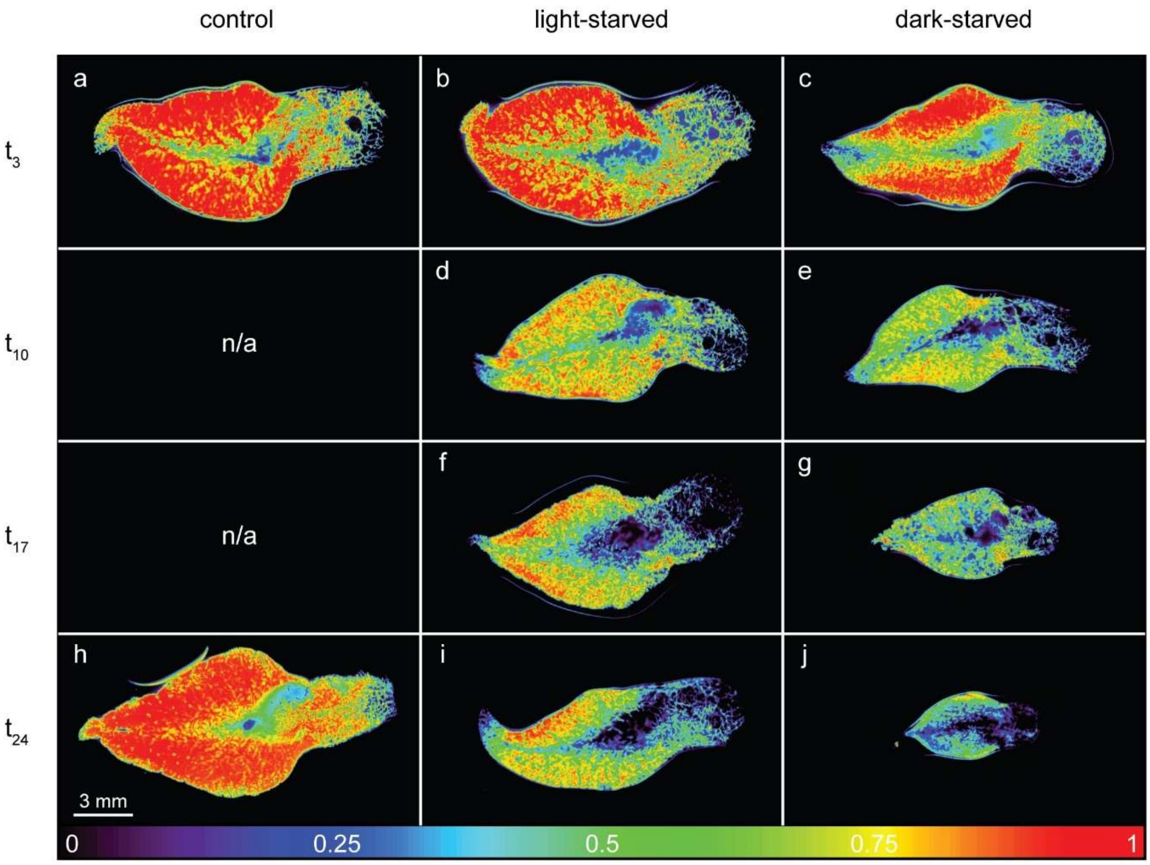

Supplementary Figure S1. **Hyperspectral imaging of *Elysia viridis*.** Normalized difference vegetation index (NDVI) images of animals feeding on *Codium tomentosum* (Control; a, h) and starved under 14:10 h light:dark cycles (light-starved; b, d, f, i) and continuous darkness (dark-starved, c, e, g, j). The number of treatment days is represented as t_{days}. n/a: not available.

		**Chl *a***	**Chl a/Chl *b***	**Carot/Chls**
**control**	t_0	0.72 ± 0.12	2.03 ± 0.06	0.38 ± 0.08
	t_{24}	0.56 ± 0.17	1.86 ± 0.08	0.33 ± 0.14
**light-starved**	t_3	0.45 ± 0.11	1.92 ± 0.04	0.36 ± 0.14
	t_{10}	0.46 ± 0.04	1.95 ± 0.03	0.37 ± 0.11
	t_{17}	0.36 ± 0.05	1.93 ± 0.03	0.37 ± 0.11
	t_{24}	0.42 ± 0.09	1.99 ± 0.03	0.43 ± 0.05
**dark-starved**	t_3	0.55 ± 0.13	1.95 ± 0.04	0.35 ± 0.09
	t_{10}	0.41 ± 0.07	1.93 ± 0.04	0.44 ± 0.10
	t_{17}	0.11 ± 0.07	1.79 ± 0.06	0.49 ± 0.18
	t_{24}	0.10 ± 0.02	1.82 ± 0.07	0.64 ± 0.18

Supplementary Table S1. **Chlorophyll *a* (Chl *a*) concentrations and pigment ratios in *Elysia viridis*.** Chl *a* (mg g^{-1} fw), Chl *a* per chlorophyll *b* (Chl *a*/Chl *b*) and total carotenoids per chlorophylls (Carot/Chls) ratios (mean ± standard deviation, n=3) in animals feeding on *Codium tomentosum* (control), starved under a 14:10 h light:dark cycle (light-starved) and continuous darkness (dark-starved). The number of treatment days is represented as t_{days}.
